# Volumetric stratification of cT4 stage head and neck cancer

**DOI:** 10.1007/s00066-013-0413-3

**Published:** 2013-09-05

**Authors:** G. Studer, C. Glanzmann

**Affiliations:** Department of Radiation Oncology, University Hospital Zurich, Raemistr. 100, 8091 Zurich, Switzerland

**Keywords:** Volumetric staging, cT4 stage tumors, Head and neck neoplasms, Neoplasm staging, Prognosis, Volumetrisches Staging, cT4-Tumorstadium, Kopf-Hals-Tumore, Tumorstaging, Prognose

## Abstract

**Background:**

Locoregionally advanced stage head and neck cancer (HNC) is known for unfavorable outcome with only ~ 40–50 % 3-year overall survival (OS). Clinical T4 stage includes a wide range of tumor burden. The lack of further nonsurgical subgrouping of cT4 stage makes intercenter outcome of irradiated cohorts difficult. Aim of this analysis was to further stratify cT4 stage HNC using volumetric staging.

**Material and methods:**

Between January 2002 and January 2013, a total of 201 cT4 stage squamous cell cancer (SCC) HNC patients referred to our center for curative definitive radiation were consecutively irradiated. Radiation was performed using modulated techniques. Total gross tumor volumes (tGTV: primary + nodal tumor volume) of all patients have retrospectively been stratified using a prospectively evaluated volumetric staging system which bases on 3 cut-offs (15/70/130 ml), translating into 4 prognostic subgroups [V1: 1–15 ml (n = 15), V2: 16–70 ml (108), V3: 71–130 ml (62), V4: > 130 ml (16)]. OS, disease-free survival (DFS), locoregional control (LRC), and distant metastasis-free survival (DMFS) rates were calculated.

**Results:**

The mean/median follow-up was 31/23 months (range 1–116 months). The 3-year OS, DFS, LRC, and DMFS rates of the entire cohort were 63, 44, 48, and 77 %, respectively. Volumetric staging revealed its potential to prognostically statistically significantly divide the cT4 cohort into 4 volume subgroups (V1/2/3/4): OS: 90 %/72 %/58 %/18 %; DFS: 83 %/50 %/39 %/10 %; LRC: 81 %/53 %/47 %/15 %; DMFS: 93 %/90 %/70 %/41 %, all p < 0.0001.

**Conclusion:**

Volumetric staging allowed a highly statistically significant stratification of cT4 HNC stages into prognostic subgroups, which offers the chance of better intercenter comparability of irradiated advanced stage HNC cohorts.

## Abstract (de)

**Hintergrund:**

Lokoregionär fortgeschrittene Kopf-Hals-Tumoren (KHT) haben eine schlechte Prognose mit nur ~ 40–50 % 3-Jahres-Gesamtüberleben (GÜ). cT4-Stadien beinhalten eine große Spanne von Tumorvolumina. Das Fehlen einer weiteren nichtchirurgischen Unterteilung von cT4-Stadien macht den Vergleich der Resultate bestrahlter Kohorten aus verschiedenen Zentren schwierig. Ziel unserer Arbeit war, cT4-Stadien bei definitiv bestrahlten KHT-Patienten mittels volumetrischem Staging zu stratifizieren.

**Material und Methodik:**

Zwischen Januar 2002 und Januar 2013 wurden uns 201 KHT-Patienten mit einem Plattenepitelkarzinom im Stadium cT4 zur kurativen definitiven Radiotherapie zugewiesen. Alle Patienten wurden mit modulierten Techniken bestrahlt. Das Gesamttumorvolumen (tGTV: Primärtumor + Lymphknotenmetastasen) aller Patienten wurde retrospektiv mittels eines prospektiv getesteten volumetrischen Staging-Systems mit 3 Schnittwerten (15/70/130 ml) stratifiziert, was zu 4 prognostischen Subgruppen führt [V1: 1–15 ml (n = 15), V2: 16–70 ml (n = 108), V3: 71–130 ml (n = 62), V4: > 130 ml (n = 16)]. GÜ, krankheitsfreies Überleben (KFÜ), lokoregionäre Kontrolle (LRK) und metastasenfreies Überleben (MFÜ) wurden berechnet.

**Ergebnisse:**

Die mittlere/mediane Bobachtungszeit betrug 31/23 Monate (Spanne 1–116 Monate). Das 3-Jahres-GÜ, -KFÜ, -LRK und -MFÜ der gesamten Kohorte betrug 63, 44, 48 und 77 %. Mittels volumetrischem Staging konnte die cT4-Kohorte in 4 statistisch hochsignifikant unterschiedliche prognostische Untergruppen stratifiziert werden (jeweils V1/2/3/4): GÜ: 90 %/72 %/58 %/18 %; KFÜ: 83 %/50 %/39 %/10 %; LRK: 81 %/53 %/47 %/15 %; MFÜ: 93 %/90 %/70 %/41 %, alle p < 0,0001.

**Schlussfolgerung:**

Volumetrisches Staging erlaubte eine statistisch hochsignifikante Stratifizierung in prognostisch unterschiedliche Untergruppen, was eine bessere Vergleichbarkeit von Resultaten verschiedener Zentren nach primärer intensitätsmodulierter Strahlentherapie (IMRT) von cT4 KHT ermöglichte.

Advanced stage head and neck cancer (HNC) is known for generally unfavorable outcome with only ~ 40–50 % 3-year overall survival [1, 2, 3]. Clinical T4 stage includes a wide range of tumor volumes. The lack of further nonsurgical subgrouping of cT4 stage makes intercenter comparison of outcome results in irradiated cT4 patient cohorts difficult. The estimation of operability (cT4a versus cT4b) is sometimes quite dependent of a surgeon’s individual opinion and experience. In addition, the in- or exclusion of very advanced cT4 any NM0 into curatively aimed treatment regimens remains quite subjective.

The aim of this analysis was to further stratify cT4 stage squamous cell HNC disease using volumetric staging. This was performed with the help of a formerly prospectively tested and published volumetric scoring system [4, 5, 6, 7]. Using this scoring system, we previously demonstrated that volumetric staging was superior compared to the standard TN/AJCC systems regarding predictive power of disease control and survival of our irradiated cohorts.

Included in the presented analysis were all cT4 stage primary squamous cell cancer (SCC) HNC patients referred for definitive radiation.

## Methods

Between January 2002 and January 2013, a total of 201 cT4 stage SCC HNC patients were referred to our department. All were treated with curative intent with modulated radiotherapy ± chemotherapy. All patients were retrospectively stratified using a prospectively evaluated volumetric staging system. T4 lymphoepithelial nasopharynx tumors (n = 13) and paranasal tumors (n = 8) were excluded. The used staging system is based on three cut-offs (15/70/130 ml, see also previous publications [4, 5, 6, 7]) to stratify the total gross tumor volumes (tGTV: primary and nodal tumor volume), allowing a subdivision of cT4 stages into 4 prognostic subgroups [1–15 ml (n = 15), 16–70 ml (n = 108), 71–130 ml (n = 62), > 130 ml (n = 16)]. Overall survival (OS), disease-free survival (DFS), locoregional control (LRC), and distant metastasis-free survival (DMFS) rates were calculated using Kaplan–Meier curves. Demographic data and tumor characteristics are listed in Tab. 1.

**Tab. 1:** Patient and tumor characteristics

Parameters			cT4
**Patients (n)**			201
**Gender** (female:male)			25 %:75 %
**Mean age** (range)			62 (38–91) years
**Mean/median folllow-up** (range)			31/23 (1–116) months
**Histology**	Squamous cell carcinoma		201
**Diagnosis**	Mesopharynx		116 (58 %)
	Hypopharynx		42 (21 %)
	Oral cavity		24 (12 %)
	Larynx		19 (9 %)
**N stage**	N0		43 (21 %)
	N1–2b		61 (30 %)
	N2c		88 (44 %)
	N3		9 (5 %)
**Total gross tumor volume (tGTV)**	Mean	Range	64 ml (7–216)
	V1	1–15 ml	15 (7 %)
	V2	16–70 ml	108 (54 %)
	V3	71–130 ml	62 (31 %)
	V4	> 30 ml	16 (8 %)
**Concomitant systemic therapy**	None		31 (15 %)
	Cisplatin only		112 (56 %)
	Cetuximab only		25 (12 %)
	Cisplatin switched to cetuximab		33 (16 %)
**Induction chemotherapy**			36 (17 %)

All patients underwent modulated radiation therapy using simultaneously integrated boost techniques [SIB-IMRT/SIB-volumetric modulated arc therapy (SIB-VMAT)]. In 84 %, concomitant cisplatin chemotherapy (40 mg/m^2^/radiation week) or cetuximab (loading dose 400 mg/m^2^, followed by concomitant doses of 2250 mg/m^2^/radiation week) was administered. In 36 patients with very advanced disease of questionably curable stage, TPF (docetaxel, cisplatin, 5-fluorouracil)-based induction chemotherapy was given as a decision aid to add or not curatively intended radiation. The remaining 16 % of patients were treated with radiation only because of age or substantial comorbidity.

All GTVs were contoured or reviewed by at least one of the authors on all relevant axial computerized images without using interpolation; in most cases the contouring was also reviewed by a third staff physician. In addition, the wide volumetric ranges (cut-offs 15/70/130 ml) render the system quite robust with respect to interindividual contouring differences. Volumetric three-dimensional measurements (cm^3^) of contoured structures were calculated by the Varian Treatment Planning System volume algorithm (Eclipse® External Beam Planning System, Version 7.3.10 and PRO 8.9, AAA 8.9, Varian Medical Systems). A detailed description of the applied SIB modulated techniques and contouring of gross tumor volume (GTV) and planning target volumes (PTVs) has formerly been published [7]. In several patients with very large GTVs, dose compromises were performed delivering 66–68 Gy to the boost volume, while the 70 Gy dose volume was limited to the GTV.

## Statistical analysis

Statistical calculations were performed using the statistics program implemented in StatView® (version 4.5; SAS Institute, Cary, NC, USA). Univariate analyses were performed with a Cox proportional hazards regression model in StatView®. Actuarial survival data were calculated using Kaplan–Meier curves and log-rank tests implemented in StatView®. P values < 0.05 were considered statistically significant.

## Results

### Outcome prediction by volumetric scoring

Between January 2002 and January 2013, a total of 201 cT4 stage SCC HNC patients were curatively treated at our department. The mean/median follow-up was 31/23 months (range 1–116 months). In all, 67 % of all patients were alive at last follow-up, and 49 % had no signs of disease. Of the 33 % of patients who had died, 24 % died due to disease-related reasons. The 3-year OS, DFS, LRC, and DMFS rates of the entire cohort were 63, 44, 48, and 77 %, respectively.

**Fig. 1:**
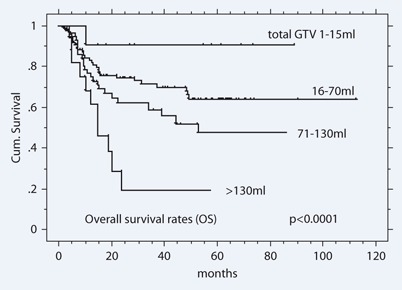
Overall survival rates of 201 cT4 patients staged by total tumor volume (ml)

Volumetric staging revealed its potential to prognostically statistically significantly divide the cT4 cohort into 4 volume subgroups (V1/2/3/4): OS: 90 %/72 %/58 %/18 %; DFS: 83 %/50 %/39 %/10 %; LRC: 81 %/53 %/47 %/15 %; DMFS: 93 %/90 %/70 %/41 %, all p < 0.0001, (Tab. 2, Fig. 1).

**Tab. 2:** Outcome according to volume subgroups (V1-4, using cut-off values of 15/70/130 ml)

			3-year survival rates			
			LRC	DMFS	DFS	OAS
cT4		n (%)	%	%	%	%
V1	1–15 ml	15 (7 %)	81	93	83	90
V2	16–70 ml	108 (54 %)	53	90	50	72
V3	71–130 ml	62 (31 %)	47	70	39	58
V4	> 130 ml	16 (8 %)	15	41	10	18
**P value**			**< 0.0001**	**< 0.0001**	**< 0.0001**	**< 0.0001**

*LRC* locoregional control rate, *DMFS* distant metastasis-free survival rate, *DFS* disease-free survival rate, *OS* overall survival rate.

### Additional parameters with potential impact on disease control and OAS

The following parameters were tested in univariate analysis:

- histopathological grading (grade 2 versus 3, no grade 1 cases), not significant,
- age (>/< 70 years), not significant,
- cT4a versus cT4b: in 63 % of the cases this differentiation was not indicated; most of the remaining cases were scored as cT4a (therefore statistically not evaluable),
- nodal status (cN0 vs N1 vs N2a vs N2b vs N2c vs N3; cN0 vs N1–2b vs N2c vs N3; cN0 vs cN1–2 vs cN3), not significant,
- systemic therapy: as the sample sizes of the subgroup with versus without systemic therapy was unbalanced (84 % vs 16 %—not the same patients with respect to substantial comorbidity and age), and systemic therapy was not homogeneous, no reliable information can be drawn from this analysis, which, however, showed a significant difference in favor of the combined modality subgroup (p = 0.2; OS 65 % vs 50 % at 3 years).

### Treatment tolerance

With respect to treatment tolerance, the following findings in 117 locoregionally controlled patients were stated as based on the last clinical visit: 16/117 patients experienced any late term grade 3/4 side effects (LENT-SOMA, 14 %). Only 6/16 patients (38 %; 3 % of all patients) suffered from persistent late term sequelae (1 × xerostomia G3, 1 × loss of taste G3, 1 × chondronecrosis, 1 × dysphagia G3, 2 × feeding tube dependence).

## Discussion

Aim of this work was to assess the potential of volumetric stratification of our cT4 SCC HNC cohort into different prognostic subgroups. We found volumetric stratification highly statistically significant in predicting outcome for different volume subgroups in the assessed cT4 HNC cohort. The volumetric system itself is considered robust with respect to interobserver GTV contouring, as its cut offs values differ markedly (15 ml/70 ml/130 ml) [4, 7]. The potential benefit of the assessed stratification lays in its more precise prediction of disease control in irradiated cT4 patient cohorts, and therefore more accurate characterization of cT4 cohorts for intercenter comparison purposes.

A weakness of this study is its retrospective stratification approach, which however applied a prospectively tested staging system [4, 5, 6, 7]. In addition, the assessed cohort includes different unbalanced tumor sites as well as unbalanced volume subgroups (Tab. 1).

To our knowledge there are no similar comparable volumetric staging analyses published. Most published volumetric focused outcome analyses were based on dichotomizing the GTV (i.e., using just one cut-off), (Tab. 3, [4, 7, 8, 9, 10, 11, 12, 13, 14, 15, 16, 17, 18, 19, 20, 21, 22, 23, 24, 25, 26, 27, 28, 29, 30, 31, 32, 33, 34, 35]). Four [17, 18, 20, 25] of the 31 listed reports were based on two or three cut-off values, our own system included. All but two analyses showed significant difference in outcome between larger vs smaller tumor volumes. Been et al. [34] failed to demonstrate statistical significance between pGTV and locoregional outcome, perhaps due to not considering the nodal tumor volume which may significantly impact locoregional outcome. Mendenhall et al. [8] found no outcome difference in tumors of the hypopharynx/base of tongue/posterior tonsillar pillar when using a cut off value of 6 ml. This cut-off may have been too low.

**Tab. 3:** Literature on head and neck cancer (HNC) outcome prediction based on volumetric classifications

Author [ref]	Year	HNC entity	Number	T	Treatment	RT technique	Mean PGTV (ml)	Cut-off value (ml)	p value LC		p value OS		
Mendenhall et al. [8]	2003	Soft pal/supragl/glottic/tonsil ant pilar	12/114/55/37	T1-4	RT(-CT)	3DCRT	5/12/8/3/12	6		< 0.05		Nl	
Mendenhall et al. [8]	2003	Hypo/BoT/tonsil post pilar	45/72/69	T1-4	RT(-CT)	3DCRT	6/24/18	6		NS		Nl	
Pameijer et al. [9]	1998	Pyrifrom sinus	23	T1/2	RT	3DCRT	Nl	6.5		0.021		Nl	
Keberle et al. [10]	2004	Hypo	45	T1-4	S(-RT)	3DCRT	8.1	8.1		0.004		Nl	
Tsou et al. [19]	2006	Hypo	51	III–IV	RT-CT	3DCRT	Nl	19		< 0.001		0.036	
Chen et al. [21]	2009	Hypo	76	III–IV	RT-CT	3DC + **IMRT**	33.4	30		< 0.0001		Nl	
Grabenbauer et al. [12]	1998	OC/Oro/hypo/larynx	87	III–IV	RT(-CT)	3DCRT	Median 110	110		Nl		0.0001	
Rudat et al. [13]	1999	OC/Oro/hypo/larynx	68	T2-4	RT-CT	3DCRT	Median 112 TGTV	112		0.0008		Nl	
Plataniotis et al. [11]	2004	OC/Oro/hypo/larynx	101	III–IV	RT(-CT)	3DCRT	17/13/22.6/14.8 median TGTV	22.8		Nl		0.01	
Strongin et al. [20]	2012	Oro/hypo/larynx	78	T1-4	RT-CT	3DC + **IMRT**	38.7	35		Nl		< 0.001	
Freeman et al. [15]	1990	Supraglottic	31	T1-4	RT	3DCRT	Nl	6		0.038		Nl	
Mukherji et al. [14]	2000	Supraglottic	37	T1-4	S(-RT)	3DCRT	9.3	16		0.04		Nl	
Gilbert et al. [16]	1987	Larynx	37	T2-4	RT	3DCRT	21.8* vs 8.9*	–		Nl		0.02	
Lee et al. [23]	1993	Glottic	29	T3	RT	3DCRT	Nl	3.5		0.02		Nl	
Pameijer et al. [24]	1997	Glottic	42	T3	RT	3DCRT	Nl	3.5		0.0002		Nl	
Hamilton et al. [18]	2004	Larynx	47	T2-3	RT	3DCRT	3.5	3 (glottic:1)		0.003		Nl	
Chua et al. [25]	1997	NPC	290	T1-3	RT(-CT)	3DCRT	6.9/18.8/52.4 in T1,2,3	20/> 60		< 0.05		Nl	
Lee et al. [17]	2008	NPC	66	T1-4	RT(-CT)	3DCRT	19.5	12.5/25/50		Nl		0.02	
Nathu et al. [26]	2000	Oro	114	T2-4	RT(-CT)	3DCRT	6.8/14.8/42.6 in T2,3,4	Nl		NS		Nl	
Hermans et al. [28]	2001	Oro	112	T1-4	RT	3DCRT	3.1/10.6/14.5/44.9 in T1-4	6/14.5/31		0.047		NS	
Keberle et al. [27]	2003	Oro	80	T1-4	S(-RT)	3DCRT	Median 4.7	Nl		NS		Nl	
Chao et al. [11]	2004	Oro	31	I–V	RT(-CT)	**IMRT**	30.5	Nl		0.05		Nl	
Been et al. [34]	2008	Oro	79	T1-4	RT(-CT)	3DC + **IMRT**	13.1	13.1		0.6 LRC		Nl	
Chung et al. [35]	2009	Oro	42	T1-4	RT±S	3DCRT	NI	35		NI		0.05	
Studer et al. [7]	2012	Oro	277	T1-4	RT(-CT)	**IMRT**	50.5 (totalGTV)	15/70/130		< 0.0001 LRC		< 0.0001	
Lok et al. [33]	2012	Oro	340	T1-4	RT-CT	**IMRT**	42.5	32.8		0.004		< 0.0001	
Johnson et al. [31]	1995	All	51	Advanced	RT	3DCRT	Median 35 TGTV	35		< 0.0001		Nl	
Doweck et al. [30]	2002	All	64	III–IV	RT-CT	3DCRT	35.4	19.6		Nl		0.0018	
Kurek et al. [32]	2003	All	107	T1-4	RT(-CT)	3DCRT	Median 32.5 and 44.4	Nl		Nl		0.02	
Studer et al. [4]	2007	All but larynx	172	T1-4	RT(-CT)	**IMRT**	37.7	15/70		< 0.02		Nl	
Hoebers et al. [22]	2008	All but NPC	46	T3-4 (92 %)	RT-CT	3DCRT	28	23		0.036 LRC		0.045	
Present work	2013	All but LE NPC	201	T4	RT(-CT)	**IMRT**	64 (total GTV)	15/70/130		< 0.0001 LRC		< 0.0001	

*Soft pal*  soft palate, *ant/post tons pil*  anterior/posterior tonsillar pillar, *hypo*  hypopharyngeal tumor, *OC*  oral cavity tumor, *oro* oropharyngeal tumor, *LE NPC*  lymphoepithelial nasopharyngeal tumor, *RT*  radiotherapy, *CT*  chemotherapy, *3DCRT*  three-dimensional conventional radiotherapy, *IMRT*  intensity-modulated radiation therapy, *PGTV*  primary gross tumor volumes, *NI* not indicated, *TGTV*  total gross tumor volume, *LC*  local control, *OS*  overall survival.

The data presented here are derived from a cohort treated with IMRT techniques, with previous careful staging (in most cases using PET-CT) [36, 37].

## Conclusion

Volumetric staging was shown to allow for highly statistically significantly stratification of cT4 stage SCC HNC into different prognostic subgroups, offering the option of better comparability of irradiated advanced stage HNC cohorts.
